# Data on periodontal health among elderly people in Bushland, Jharkhand, Magadha and Patna, India

**DOI:** 10.6026/97320630017053

**Published:** 2021-01-31

**Authors:** Silpi Chatterjee, Minti Kumari, Anurag Rai, Tanoj Kumar, Akash Tripathi, Shruti Gupta

**Affiliations:** 1 Department of Public Health Dentistry, Hazaribag College of Dental Sciences and Hospital, Jharkhand, India; 2 Department of Public Health Dentistry Government Dental College and Hospital, Patna, India; 3 Department of Orthodontics and Dentofacial Orthopaedics Government Dental College and Hospital, Patna, India; 4 Department of Oral Pathology Government Dental College and Hospital,Patna, India; 5 Department of Periodontology. Hazaribag College of Dental Sciences & Hospital, Jharkhand, India

**Keywords:** Oral cleanliness, periodontal health, pathogenic organism, PI scores

## Abstract

It is of interest to report data on periodontal Health among elderly people in Bushland, Jharkhand, Magadha and Patna, India. The sample comprised of a 130 elderly people. The studies device comprised of a semi-structured survey with thirteen questions. Data shows that old people in Jharkhand suffered from advanced periodontal ailment (47.6%) with easy gingivitis (33.8%). Data also shows that grownups (88.2% grownup males, 64.5% girls in Jharkhand and 34.5% grownup males and 88.9% girls in Bihar) used toothpaste and toothbrush as their primary style for tooth cleansing. These data help in providing improved dental service to rural population in India.

## Background

Suitable oral cleanliness remains deliberated to be a vital difficulty for periodontal condition, and the maximum suitable oral cleanliness dependency is commonplace and correct enamel brushing skill and usage of supplementary oral cleanliness aids [3, 7,4]. Several researches have shown an intense lower in gingival infection and concise complexities with development in oral cleanliness [6]. Therefore, it's far crucial to invent on each day's oral cleanliness methods. Carelessness in preserving oral healthiness could drive bacterial construct-up and plaque foundation, pavement the manner for pathogenic bacterial classes related to extreme types of periodontal ailments [1,2,6,13]. Populace research offers sturdy proof of inter-relationship among dental plaque and the improvement of gingivitis, consequently accompanied in using manner of the periodontal breakdown [1,3,9]. Periodontal illnesses are some of the maximum accepted persistent illnesses affecting people of all age agencies universally [10,11,8]. Despite the fact that the sickness occupies a widespread fraction of the prevailing oral illnesses, much less interest has been given to evaluate the hazard elements and occurrence in populations at big [10,5,12]. Consequently investigating the threat elements and symptoms could advantage in retaining oral healthiness and prevent the improvement of any practice of periodontal illness. Generally, related elements consist of negative oral and dental fitness, terrible food plans, behavior, and primary systemic conditions [6,1,10]. Precaution ary events are focused at putting off the aspects related with plaque creation that deteriorates tissue resistance. Research and inspections depict that there's a marked laxity of dental interest among the Indian populace [12]. A reduced amount of one third complies with proper oral cleanliness measures frequently resulting in numerous periodontal conditions [12].

## Methodology

### Dataset:

The sample comprised of a 130 elderly people. The teeth cleaning materials include (1) tooth brush; (2) tooth paste; (3) Tooth powder; (4) finger; (5) neem stick; (6) coal powder; (7) tobacco and (8) any other.

### Model:

The studies device comprised of a semi-structured survey with thirteen questions as described by AL Russell (1956).

### Analysis:

Data was analyzed using the Statistical Package for Social Sciences (SPSS), model 23 (SPSS Inc., Chicago IL). Descriptive data had been calculated. A Chi-square takes a look at turned into implemented to decide the association between oral cleanliness follows and related periodontal situations.

## Results:

The data consisted of 130 grownups, 34 men and 31 females from Jharkhand and 29 men, 36 girls from Bihar. The sample distribution in accordance to mean age and gender is shown in Table 1(see PDF). About 3% of the grownups in Bihar and 1.5% populace in Jharkhand mentioned a clinically healthy periodontium. The highest percentage of the pattern grownup population in Jharkhand suffered from advanced periodontal disorder (47.6%). In contrast, in Bihar, the score was better with simple gingivitis (33.8%) (Table 2 - see PDF), and the association between the two populations become statistically significant. The grownups (88.2% men, 64.5% females in Jharkhand and 34.5% males and 88.9% girls in Bihar) used toothpaste and toothbrush as their primary mode for enamel cleansing (Table 3 - see PDF), and their corresponding implies PI rankings had been enormously decreasing when it comes to other methods of teeth-cleansing said. As shown in Table 4 (see PDF), the frequency of grownups the use of a gentle toothbrush in the kingdom of Bihar was 20.7% males, seventy two.7% ladies and in Jharkhand was 50% males, 38.7% women. The mean PI rating related to the normal use of a smooth toothbrush becomes a decrease in the usage of a harsh toothbrush. On the country degree, the attention to emphasize different aspects even as buying a toothbrush is depicted in Table 5(see PDF). Table 6(see PDF) affords the sample distribution in step with the frequency to trade the toothbrush and the related mean PI score. Both states recorded an appreciably better rating where the question becomes unanswered, declaring a lack of expertise in the issue count number. Affiliation is proved to be statistically widespread. Consistent with the frequency of teeth brushing and associated mean PI ratings, it becomes discovered that two times an afternoon brushing addiction recorded an exceedingly lower suggest score in each state than brushing once a day. The proportion population with twice an afternoon brushing changed into 61.4%( 32.4% grownup males, 29% females) in Jharkhand and 45.8% (6.9%males and 38.9% girls) in Bihar respectively.

Alternatively, the variation in mean rankings extracted from the pattern on the idea of the duration of teeth brushing (Table 7 - see PDF) in both states proved to be statistically insignificant. The data distribution in Table 8, 55.9% of grownup males, 35.5% of women in Jharkhand, and 13.8% grownup males, 19.4% of females in Bihar had been aware of diverse reasons to trade a toothbrush. The relative PI scores recorded in those topics distinctly decreased from the relaxation of the sample population. Table 9 (see PDF) shows the distribution of grownups in keeping with present habits. The mean PI rating in grownups with no follows was significant lower. The sample distribution, according to reviling systemic conditions (Table 10 - see PDF), recorded excessive implies PI ratings in subjects suffering from chronic ailments like diabetes and hypertension than in clinically healthiness of people. This distribution changed into statistically full-size.

## Discussion:

47.6% of grownups from Jharkhand, and 23% of grownups from Bihar showed the presence of excellent periodontal sickness conditions. Green recurring oral cleanliness follows (correct method, frequency, and duration of tooth brushing, awareness approximately the dental merchandise, loss of conduct) had been related to an exceedingly lower mean PI score. A 12 months-old look performed with the aid of Wairau group [2] on personnel of a manufacturing unit in Oslo (>800 subjects, 20-59 years age) recorded 60% improvement in periodontal fitness and a 50% reduction in teeth loss after development in oral cleanliness conditions (careful technique closer to correct brushing strategies, interdental aids). Oral healthiness surveys conducted in Burkina Faso, Africa, discovered comparable findings [5]. They showed the prevalence of advanced periodontal ailments in grownups with poor oral cleanliness. Loe et al. [9] have proven a giant regression in gingival irritation to publish the right oral cleanliness intervention [2]. Findings from a comparable clinical trial propose that everyday tooth brushing prompted marked reduction inside the inflammatory situations of the gingival [8]. Concerning the accumulated records, it may be advised that our survey confirmed the preceding observation of a uniform affiliation of poor oral healthiness with the prevalence of periodontal conditions. Additionally, the existing statistics disclosed a lousy effect of current habits and systemic situations on periodontal healthiness. The suggested PI rating recorded became lower among individuals who abstained from all conduct and practiced a healthiness way of life. The consequences come in agreement with a report submitted by the WHO on the prevention of periodontal ailments. Findings from comparable studies concluded a strong affiliation of smoking and tobacco addiction with periodontal attachment loss [10]. In line with the mounted studies, warmness from smoking and nicotine in tobacco impairs recuperation and enhances attachment loss inflicting periodontal breakdown [2]. The regular use of tobacco and other harmful materials in any form could affect the immune system in the long run and decrease down the host resistance paving the manner to multiple oral and systemic conditions [10]. Association of systemic conditions with a periodontal breakdown is a well-documented reality. Research has concluded more incidence and rapid progression of periodontal situations in individuals with diabetes mellitus [11]. In short, the survey indicated that the superiority of desirable oral cleanliness associated with the relatively healthy periodontium. The need for oral fitness care and oral cleanliness awareness turned into evidence inside the people. But, no assessment was recorded for the plaque and calculus in the pattern population. Plaque performs an essential function within the development of the periodontal disorder [1,2,8].
